# Power-Doppler-based NH002 microbubble sonoporation with chemotherapy relieves hypoxia and enhances the efficacy of chemotherapy and immunotherapy for pancreatic tumors

**DOI:** 10.1038/s41598-024-54432-y

**Published:** 2024-06-03

**Authors:** Sheng-Yan Wu, Chung-Hsin Wang, Shih-Tsung Kang, Ching-Fang Yu, Fang-Hsin Chen, Chi-Shiun Chiang

**Affiliations:** 1 Department of Biomedical Engineering and Environmental Sciences, National Tsing Hua University, Hsinchu, 30013 Taiwan; 2 Trust Bio-Sonics, Inc., Zhubei, 30261 Taiwan; 3 Research Center for Radiation Medicine, Chang Gung University, Taoyuan, 33302 Taiwan; 4 Department of Radiation Oncology, Chang Gung Memorial Hospital Linkou Branch, Taoyuan, 33382 Taiwan; 5 Institute of Nuclear Engineering and Science, National Tsing Hua University, Hsinchu, 30013 Taiwan; 6 The BNCT Research Center, National Tsing Hua University, Hsinchu, 30013 Taiwan

**Keywords:** Cancer microenvironment, Pancreatic cancer, Cancer immunotherapy, Preclinical research

## Abstract

Pancreatic ductal adenocarcinoma (PDAC) poses challenges due to late-stage diagnosis and limited treatment response, often attributed to the hypoxic tumor microenvironment (TME). Sonoporation, combining ultrasound and microbubbles, holds promise for enhancing therapy. However, additional preclinical research utilizing commercially available ultrasound equipment for PDAC treatment while delving into the TME's intricacies is necessary. This study investigated the potential of using a clinically available ultrasound system and phase 2-proven microbubbles to relieve tumor hypoxia and enhance the efficacy of chemotherapy and immunotherapy in a murine PDAC model. This approach enables early PDAC detection and blood-flow-sensitive Power-Doppler sonoporation in combination with chemotherapy. It significantly extended treated mice's median survival compared to chemotherapy alone. Mechanistically, this combination therapy enhanced tumor perfusion and substantially reduced tumor hypoxia (77% and 67%, 1- and 3-days post-treatment). Additionally, cluster of differentiation 8 (CD8) T-cell infiltration increased four-fold afterward. The combined treatment demonstrated a strengthening of the anti-programmed death-ligand 1(αPDL1) therapy against PDAC. Our study illustrates the feasibility of using a clinically available ultrasound system with NH-002 microbubbles for early tumor detection, alleviating hypoxic TME, and improving chemotherapy and immunotherapy. It suggests the development of an adjuvant theragnostic protocol incorporating Power-Doppler sonoporation for pancreatic tumor treatment.

## Introduction

PDAC has been one of the deadliest cancers over the last ten years, with an estimated 4.2% of 5-year survival rate among all stages of patients^1^. The incidence rate is still rising and is predicted to come to the forefront in 2030^2^. Most newly diagnosed PDAC patients already have locally advanced tumors or distant metastasis considered surgically unresectable^3^. After the diagnosis, these patients have only 4–6 months of survival^4^. Currently, chemotherapy is the primary treatment against pancreatic cancer, as radiation therapy is rarely used due to its proximity to the gastrointestinal tract^5^. For chemotherapy, gemcitabine and 5-Fluorouracil (5-FU) are the front-line drugs used through the past two decades. With more patient tolerance, gemcitabine has become the standard care for monotherapy, with an average survival of 6.7 months^6^. Recently, a cocktail therapy clinical trial (FOLFIRINOX) with four drugs (5-FU, irinotecan, folinic, and oxaliplatin) demonstrated an improvement of overall survival from 6.8 to 11.1 months compared to the gemcitabine only in the unresectable patients^7^. Since then, FOLFIRINOX has been the best option for metastatic PDAC patients or as adjuvant therapy in resectable patients^8^. However, despite the recommendation of different treatment regimens, the survival benefit is still minimal, and the efficacy of chemotherapy is hindered by the TME of PDAC^9^. The hypo-vascular characteristic and desmoplastic stromal cells are attributed to the hypoxic TME of PDAC, thus increasing their resistance to chemotherapy or other therapeutic strategies, including immune checkpoint blockade (ICB) therapy^10,11^. Therefore, novel treatment interventions are urgently needed by modulating the TME of PDAC to benefit chemotherapy, immunotherapy, or early diagnosis approaches.

The clinic has employed diagnostic ultrasound as a non-invasive imaging method for over 40 years, playing a vital role in detecting and staging pancreas tumors^12^. Practically, transabdominal ultrasound is often used for the initial diagnosis when the doctor suspects a tumor. The minimally invasive endoscopic ultrasound (EUS) is then employed to obtain a more precise image of the PDAC^13,14^. Contrast-enhanced ultrasound (CEUS), which effectively increases the signal-to-noise ratio of blood, has emerged as another non-invasive imaging modality for visualizing vascular dynamics^15^. CEUS utilized microbubbles containing gas within a shell. Once in the bloodstream, their high echogenicity provides a more amplified signal in the microvasculature than in the surrounding tissue^16^. With additional blood information, clinicians can depict the real-time blood perfusion of various organs, including the heart, liver, kidney, and pancreas. Pancreatic lesions can be verified and characterized more accurately with CEUS^17^.

In addition to the improved diagnosis, researchers have observed oscillations in microbubbles when exposed to ultrasound. Depending on ultrasound intensity, bubbles cavitate and undergo volumetric changes or violent expansion^18^. When this process occurs near the cell membrane or vessel wall, tiny pores can form, potentially enhancing the transport of therapeutic macromolecules. This phenomenon is called “sonoporation”. Sonoporation, under stable and inertial cavitation, has successfully enhanced chemotherapy in many preclinical cancer models^19–21^. However, most of these studies use specially designed ultrasound systems or transducers, mainly focusing on the improved uptake effect of drug molecules. Few studies emphasize the TME variation of PDAC, such as hypoxia, induced by sonoporation. Recently, researchers have started applying Power Doppler ultrasound imaging to evaluate tumor perfusion after sonoporation^22,23^.

Power Doppler is an ultrasound image technique that detects blood flow, commonly employed to examine the tumor vasculature in various cancers^24^. Compared to conventional brightness mode (B-mode), Power Doppler provides more ultrasound pulses, potentially increasing the cavitation dose and enhancing sonoporation effects. Additionally, doctors can select the region of interest using Power Doppler mode, making it a promising choice for targeted therapy^25^. Despite being predominantly used for vascularity evaluation, Power Doppler has not been explored extensively for treatment. Given its widespread availability on almost every clinical ultrasound system, we aimed to assess the feasibility of using Power Doppler-based sonoporation with commercially available diagnostic ultrasound apparatus for PDAC treatment.

In this study, we performed CEUS via NH002 (Perfluoro-propane lipid microbubbles, currently completed Phase I/II clinical trial NCT04185246 for contrast echocardiography) to diagnose the murine orthotopic pancreatic tumor, evaluate the tumor perfusion, and investigate the feasibility of using Power Doppler-based NH002 sonoporation with chemotherapy (PDNSC) and immune-checkpoint inhibitor to treat PDAC tumor.

## Method and materials

### Mice

Eight-week-old C57BL/6J male mice were purchased from the National Laboratory Animal Center of Taiwan. All animal performances followed the guidelines of the Institutional Animal Care and Use Committee (IACUC) of National Tsing Hua University, Taiwan (IACUC approval No. 109067).

### Cell line

UN-KC-6141, a murine PDAC cell line (given by Prof. Surinder K. Batra, the University of Nebraska Medical Center, Omaha, Nebraska, USA)^26^, was incubated at 37 °C, 5% CO_2_ under humid conditions. Cells were maintained in Dulbecco’s modified Eagles medium (DMEM; Gibco®, 12100046, Grand Island, NY, USA) with 10% fetal bovine serum (FBS; Gibco®, 16000044), 1% penicillin–streptomycin (PS; Gibco®, 15140122). Before utilizing the cells, Mycoplasma contamination was examined by an EZ-PCR™ Mycoplasma detection kit (Biological Industry, 20-700-20, Beit HaEmek, Israel).

### In vivo pancreatic tumor model

The orthotopic pancreatic tumor model was generated by implanting UN-KC-6141 cells into the pancreas as described in our previous publication^5^ with minor modifications. Briefly, mice were anesthetized with a 1:1 mixture of Zoletil®50 (Virbac, 7J7ZA, Carros, France) and 2% Rompun® (Bayer HealthCare Animal Health, CAPROM-L-003, Germany). A small incision was made on the left abdomen (spleen side). The spleen was dragged out along with pancreas tissue. A cell number 1 × 10^4^, in the form of a semi-solid gel spheroid, was embedded into the pancreas head. To form the semi-solid spheroid, 2 μl of 1:1 mixture with DMEM and Matrix gel (Corning, 356237, Bedford, MA, USA) were used and pumped by an automatic pump (KD Scientific, 311, Holliston, MA, USA). The wound was closely sutured and covered with ointment to avoid infection. The body weight of mice was recorded every other day after tumor inoculation. Tumor-bearing mice were euthanized after showing neurologic deficits (lethargy, failure to canter, dyspnea, and back arching).

### Pancreatic tumor size assessed by ultrasound imaging

After tumor implantation, mice were screened with ultrasound imaging on days 10, 13, 16, and 21. Briefly, mice were anesthetized, and the body hair of the observed area was entirely removed by depilation crème. Imaging was performed using a portable ultrasound system (Philips, CX50, Amsterdam, Holland) with an L12-3 linear broadband ultrasound transducer (Philips). Images were acquired with a depth of 3 cm and a mechanical index (MI) value of 0.8. The stomach and spleen were scanned and marked in the ultrasound image as a reference (Fig. S1A), followed by the pancreas and the tumor. Once the tumor was located, video (frame rate 31 Hz) was recorded to find the largest section area. Ultrasound videos and images were further processed with RadiAnt DICOM Viewer, and the total area of all tumor sections was circled and calculated using Image-Pro 6.0 software.

### CEUS PDAC characterization

Tumor-bearing mice were lying on one side for ultrasound imaging. Microbubbles (3 × 10^8^ particles in 50 μl PBS, Trust Bio-sonics, NH002, Perfluoro-propane lipid microbubbles, Hsinchu, Taiwan) were administrated via IV (intravenous) injection. Before the experiment, the microbubbles were freshly prepared with a temperature-controlled high-speed agitator (Trust Bio-sonics, Transmix). The images were acquired by the linear broadband ultrasound transducer (Philips, L12-3) in contrast imaging mode setting with a depth of 4 cm and MI value of 0.08. Live video (frame rate 19 Hz) was recorded to monitor the perfusion dynamics. The signal intensity of each image pixel was further calculated by the Image-J software.

### Sonoporation treatment protocol

Different ultrasound sonoporation treatment settings were applied for separate experiments. For the sonoporation under B-mode, mice were randomly separated into three groups: Control, Dox only, and Dox + MB (doxorubicin plus microbubble-induced sonoporation) ten days after tumor inoculation. Treatments were conducted on day 10 and day 16 for both treatment groups. Mice were positioned, and an intravenous (IV) injection of 2.5 mg/kg doxorubicin (Dox; Pfizer, ADRIAMYCIN®, New York, USA) was given to the mice 10 min before sonoporation. The tumors were screened and located right after the administration of Dox. A 50 μl IV injection of 3 × 10^8^ microbubble was then given to mice every 5 min for four consecutive times, and the MI value of the B-mode imaging was set as 0.4 throughout the treatment (Fig. S2C,D). A ring stand and a three-prong clamp held the transducer to ensure precise positioning. The transducer was moved back and forth to cover all the tumor sections during the sonoporation. The Dox-only group went through the same ultrasound scanning procedures without microbubble injection. Control mice were treated with PBS and only imaged with ultrasound to obtain the tumor image with the largest section area.

For the Power Doppler-based sonoporation, tumor location was confirmed and circled as the region of interest (ROI) by the Power Doppler mode 10 min before sonoporation. Dox (2.5 mg/kg) or gemcitabine (30 mg/kg) was administered together with microbubble. The total doses of Dox and microbubble remained the same as the previous B-mode setting. They were IV delivered with an automatic micropump (KD Scientific, KDS-310, Holliston MA, USA) at an infusion rate of 50 μl/min for 4 min. Mice in the microbubble-only group received only microbubbles. Once the infusion started, the B-mode was switched to the already ROI-selected Power Doppler mode, and the mechanical index was set to 0.4 (Fig. 2A,B). Mice were anesthetized all the time and kept warm during the treatment.

### PDNSC combined ICB therapy

Ten days after the tumor inoculation, mice were randomly separated into three groups (n > 4 for each group): anti-programmed death-ligand 1 only (αPDL1; Bio X cell, InVivoMAb anti-mouse PD-L1, BE0101, NH, USA), Dox plus αPDL1 (Dox + αPDL1), Power doppler sonoporation plus Dox as well as αPDL1 (Dox + αPDL1 + MB). The PDNSC treatment protocols were the same as in the settings mentioned above. Mice were intraperitoneally (IP) injected αPDL1 (8 mg/kg) on days 10, 13, 16, and 19.

### Tumor perfusion test after PDNSC

A tumor specimen (20 mm^3^) was dissected from the UN-KC-6141 tumor-bearing mice, and then the xenograft was implanted into the pancreas of the other healthy mouse. The following surgical protocol was the same as the orthotopic pancreatic tumor injection. After the tumor reached 50 mm^2^ under the ultrasound examination, mice were randomly divided into Dox only (mice receive Dox only) and Dox + MB (Dox plus microbubble-induced sonoporation). Tumor perfusion was examined one day before treatment and days 0, 1, 3, and 6 after. For the tumor perfusion examination, 20 μl of 1.2 × 10^8^ microbubbles were IV injected into mice. Then, the dynamic reperfusion was recorded as a video (frame rate 19 Hz) in the contrast mode by the CX50 ultrasound system following bubble flashing. The image in the video was snapped 7 s after the bubble flashing to calculate the tumor perfusion area. The percentage of tumor perfusion area was calculated from the bright tumor perfused area ((entire tumor area − non-perfused tumor area)/total tumor area) × 100%. The mean peak intensity of the tumor area during the microbubble reperfusion was calculated using Image J, and AUC (area under the curve) of time × peak intensity was calculated using the Prism software 8.0 (GraphPad, San Diego CA, USA).

### Tissue immunofluorescence analysis

The hypoxia bio-marker pimonidazole (PIMO, 160 mg/kg, HPI-100, HPI, Burlington, MA, USA) was IP injected into mice one hour before sacrifice. The tumor tissue was embedded with the Optimal Cutting Temperature (OCT) compound (Sakura, Finetek, Torrance, CA, USA) and immediately kept at – 80 °C. Frozen tissues were sliced by the cryo-microtome (Leica, CM1850, Heidelberger, Germany) with 10 μm thickness and adhered to the salinized slide (MUTO PURE CHEMICALS, 511614, Tokyo, Japan). The frozen sections were fixed with cold methanol and permeabilized with 0.1% Tween-20 (Sigma, St. Louis, MO, USA) and subsequently blocked with 4% FBS, 1% goat serum (Gibco®, 16210-064) in PBS to reduce the non-specific binding for 1 h at room temperature. First antibodies were stained as follows: purified rat anti-mouse CD31 (1:200, BD Pharmingen, 550274, San Jose CA, USA), purified rat anti-mouse CD8 (1:100, BD Pharmingen, 550281), purified rat anti-mouse CD4 (1:100, BD Pharmingen, 550280), mouse anti-mouse AE1/AE3 (1:200 Abcam, ab27988, Cambridge, UK), and mouse anti-Hypoxy biomarker (1:100, HPI, HPI-100 kit) overnight at 4 °C. Secondary antibodies conjugated with Alexa Fluor 488 goat anti-mouse (1:200 Thermo Fisher Scientific, A21121, Waltham MA, USA), Alexa Fluor 594 goat anti-rat (1: 200 Thermo Fisher Scientific, A11012), and Alexa Fluor 594 goat anti-mouse (1: 200 Thermo Fisher Scientific, A21125) were stained for 1 h at room temperature. Hochest33342 (2.5 mg/ml, Thermo Fisher Scientific, H1399) was stained to visualize the nucleus. For preservation, slices were mounted using an antifade mounting medium (VECTOR, H-1000, Burlingame, CA, USA). Images were taken by the AxioCam MCR-5 on Axiovertskop 40 microscope (Carl Zeiss, Axioskop 40 FL, Goettingen, GERMANY) and analyzed by Image-Pro 6.0 software.

### H&E staining

Methanol fixed section slides were air-dried and stained with Hematoxylin (SIG-MA, GHS232-1L) for 1 min, followed by water rinsing. The slides were treated with 0.25% Ammonia after rinsing. The air-dried slices were subsequently stained with Eosin (SIGMA, HT110116-500ML) for 20 s and mounted with VectaMount® Permanent (VECTOR, H-5000).

### Blood chemistry test

The blood (200 μl) was collected from tumor-bearing mice 24 h post-treatment to examine the acute side effects of the treatments. The whole blood was centrifuged at 2000 × g for 10 min at 4 °C. 60 μl of blood serum was collected from the supernatant and tested on the reagent disc (AmiShield, 001-3GYC, Taoyuan, Taiwan) with the biochemical analytical instrument (AmiShield, VCA-TE-300).

### In vitro cytotoxicity test

UN-KC-6141 (3.6 × 10^3^ cells) were seeded in 96-well overnight; on the other day, diluted Dox or NH002 microbubble was added to the medium and incubated at 37 °C, 5% CO2 for 24, 48, and 72 h, respectively. Following incubation, 250 μg of 3-(4,5-Dimethylthiazol-2-yl)-2,5-diphenyltetrazolium bromide (MTT, Sigma, SI-5655) was added into each well for 3 h and measured by the microplate reader (TECAN, Infinity® 200 PRO, Männedorf, Switzerland).

### Statistics

Statistical analysis was performed by the Prism software 8.0 with two-tailed Student’s t-tests, and a *P*-value < 0.05 was recognized as statistical significance.

### Ethics approval

All experiments in this study were conducted in accordance with ARRIVE guidelines, and the protocols were approved by the Institutional Animal Care and Use Committee (IACUC) of National Tsing Hua University, Taiwan (IACUC approval No. 109067).

## Results

### Contrast-enhanced ultrasound imagination reveals PDAC perfusion characteristics

The pancreatic cancer cell line, UN-KC-6141, has been reported to establish orthotopic PDAC tumors successfully^5,26^. To monitor tumor progression in the orthotopic model, diagnostic B-mode ultrasound was performed on the tumor-bearing mice to locate the pancreas and tumor by referencing the surrounding organs. (Fig. S1A, left and middle). The B-mode ultrasound images provided insights into the landscape changes of the organs as we scanned from location A to B. Specifically, the pancreas (marked with a rectangular circle in skin color) was found beneath the spleen (indicated by an irregular red circle). In contrast, the dark contour within the pancreas stated the presence of the tumor, as shown by the irregular yellow circle (Fig. S1A, right). While using reference organs to locate the pancreatic tumor allowed for a rapid examination, distinguishing between the tumor and surrounding tissue, particularly in dark areas (Fig. S1A), proved challenging. As a result, additional strategies are needed to identify PDAC tumors accurately.

**Figure 1:**
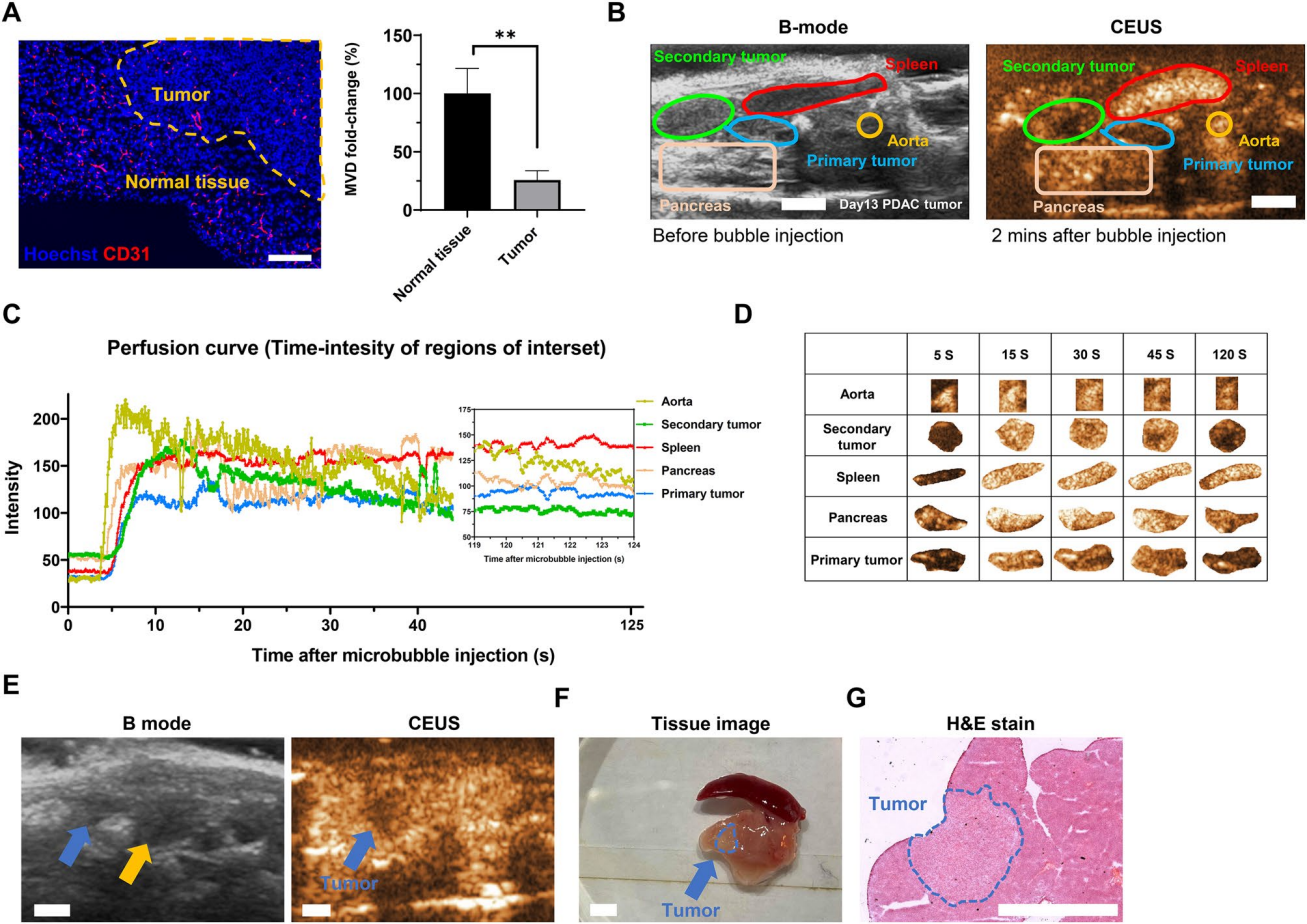
Contrast-enhanced ultrasound characterization for murine orthotopic UN-KC-6141 tumors. (**A**) Immunofluorescence staining of UN-KC-6141 tumor. Sections were stained with CD31 (red) and Hoechst33342 (blue). Tumor and normal tissue regions (yellow dashed line indicated tumor region) were separated and calculated for MVD (n = 3). A two-tailed unpaired t-test was used. **:*P* < 0.01. (**B**) The ultrasound image of the day 13 tumor-bearing mice. B-mode image (left), Contrast-enhanced ultrasound (CEUS) image (right) via NH002. Pancreas (incarnate color), Spleen (red color), Aorta (yellow color), Primary tumor (blue color), and Secondary tumor (green color). (**C**) The time-dependent intensity curve of different regions circled in (**B**) after 50 μl of 3 × 10^8^ microbubble injection. (**D**) Representative diagrams of each region at 5, 15, 30, 45, and 120 s post microbubble injection. (**E**) The ultrasound image of day 10 tumor-bearing mice, B-mode image (left, blue, and yellow arrow = suspicious area), and CEUS image (right, blue arrow = tumor). (**F**) Tissue images were taken after mice were euthanized (blue circle = tumor) after the treatment. (**G**) H & E staining of pancreas tissue with tumor (blue circle). Scale bar = 0.25 cm.

PDAC was clinically characterized as a hypovascular tumor with poor perfusion^27^. To evaluate whether the orthotopic UN-KC-6141 tumor had the same characteristics as clinical PDAC, immunofluorescence staining of vessel endothelial marker CD31 was applied. The results demonstrated that the tumor's mean vessel density (MVD) was significantly lower than the nearby normal pancreas tissue. Tumors exhibited only a quarter of the MVD shown in normal tissue (Fig. 1A), suggesting tumors might have worse perfusion than normal tissues. To examine the perfusion, CEUS imagination was performed via bolus injection of NH002 microbubbles. Based on the anatomical position, the red circle, denoted as the spleen, possessed a strong signal until 2 min after the microbubble injection, indicating high perfusion (Fig. 1B, right). On the other hand, the green and blue circles showed the least signal intensity at this time, indicating that they were the tumors (Fig. 1B right, Supplement video 1). The perfusion dynamics were recorded and plotted as an intensity curve (Fig. 1C), in which the highest signal intensity of the incoming microbubbles was from the aorta with a quick accumulation at 5 s after microbubble injection. Interestingly, although PDAC was recognized as a low perfusion tumor, it still exhibited a rapid wash-in phase around 5–15 s (green and blue line) and a clear wash-out afterward. The secondary tumor in the abdomen fluctuated more than the primary tumor, and both stayed as lower perfusion areas after 30 s compared to the spleen, as shown in the snap images of each organ (Fig. 1C,D). To validate the effectiveness of CEUS imaging, particularly for small tumor lesions, we examined a day-10 tumor. Similar to the observations on the day-13 tumor (Fig. 1B–D), suspicious regions were detected in the B-mode image (indicated by blue and yellow arrows in Fig. 1E). Using CEUS, we identified the tumor lesion with a low microbubble perfusion area (Fig. 1E, blue arrow). This finding was further confirmed through pathology and H&E staining (Fig. 1F,G).

In summary, utilizing this CEUS technique, we could successfully detect small tumors, as illustrated in Figure S1C. For example, a small PDAC tumor (highlighted by a yellow circle) was accurately identified from the image and validated through live sacrifice, H&E staining, and immunofluorescence staining using the pan-keratin marker AE1/AE3 (Fig. S1D–F). These findings demonstrated that UN-KC-6141 tumors exhibited a low perfusion tumor microenvironment, and microbubble-based CEUS could effectively distinguish tumors with a diameter less than 5 mm from normal tissues.

### Power Doppler-based sonoporation enhances the efficacy of Dox-mediated PDAC tumor growth delay

The above results indicated that CEUS could be beneficial for pancreatic tumor diagnosis. To further explore the feasibility of sonoporation using a clinical diagnostic ultrasound system combined with chemotherapy for PDAC treatment, the chemo-drug Dox was tested in cellular and in vivo studies. Dox exerted cytotoxicity on UN-KC-6141 cells with IC_50_ of 0.199, 0.013, and 0.009 μg/ml after 24, 48, and 72 h, respectively (Fig. S2A), while there was no cytotoxicity effect for the microbubble treatment alone group (Fig. S2B). The effect of Dox on tumor control was then examined by injecting Dox (2.5 mg/kg) into tumor-bearing mice on days 10 and 16 after inoculation (Fig. S2C). A total amount of 5 mg/kg of Dox was reported to be a safe dose^28,29^. Sonoporation was performed on the same day as Dox administration on days 10 and 16. The clinical program^30,31^ was modified to inject Dox 10 min before sonoporation, and NH002 microbubbles were given every 5 min for four consecutive times (Fig. S2D). The mechanical index was set at 0.4 to ensure sufficient oscillation of microbubbles while preventing bioeffects^32,33^. The results showed that two doses of Dox treatment could slightly extend the surviving days of mice-bearing UN-KC-6141 tumors from 22.0 to 24.0 days (Fig. S2E). A significant tumor growth delay was noted on day 13 and continued to day 21 (Fig. S2F,G). However, despite observing a trend of reduction in tumor size from day 16 to day 21 compared to the Dox treatment group, B-mode-based sonoporation did not improve the therapeutic efficacy of Dox. The mice survival curve showed a slight increase in median survival (24.0 vs. 26.0 days) but without a significant difference between the Dox-only group and the sonoporation plus Dox group (Fig. S2E,F,G).

**Figure 2:**
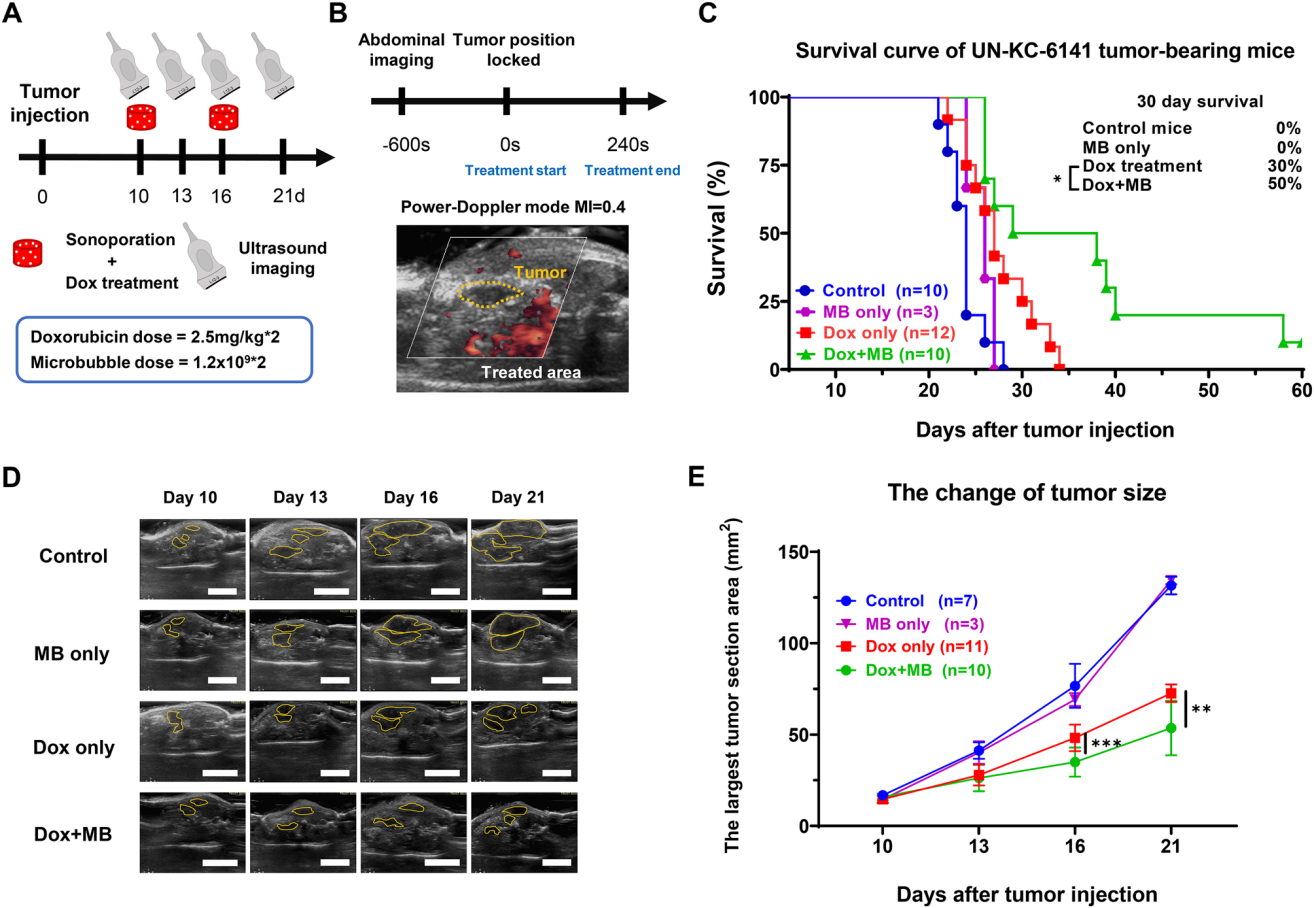
Combined doxorubicin with Power Doppler-based sonoporation slows down UN-KC-6141 tumor progression. (**A**) The schedule of the treatment protocol. (**B**) Power Doppler-based sonoporation treatment scheme. 2.5 mg/kg of doxorubicin was given together with 6 × 10^9^/ml microbubbles and delivered at 50 μl/min with an automatic pump. (**C**) Kaplan–Meier survival curve of PDAC-bearing mice, Control mice without any treatments (n = 10), MB only group received sonoporation only (n = 3). The Dox-only group received doxorubicin (n = 12), Dox + MB group received doxorubicin plus Power Doppler-based sonoporation (n = 10). (**D**) Representative figures of ultrasound images (Yellow circle indicated tumor region) at different time points. (**E**) The largest tumor section area of orthotopic UN-KC-6141 tumor examined at 10, 13, 16, and 21 post-tumor implantation. Scale bar = 1 cm. A two-tailed unpaired t-test was used to compare the tumor size at each time point between the Dox only and Dox + MB groups. *: *p* < 0.05, **: *p* < 0.01, ***: *p* < 0.001.

**Figure 3:**
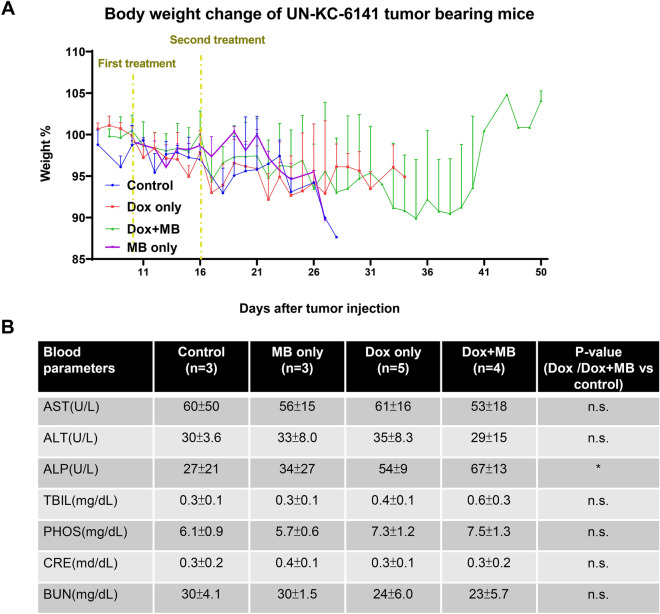
Adverse effects of mice after the treatments. (**A**) Mice body weight variation. Mice’s body weight was recorded every two to three days until sacrificing. Control mice without treatments (n = 8), MB only group received sonoporation only (n = 3). The Dox-only group received doxorubicin (n = 11), Dox + MB group received doxorubicin plus Power Doppler-based sonoporation (n = 10). (**B**) Table of Blood biochemical tests. Mice’s blood was collected 24 h after treatment for biochemical examination. Values are presented as the average ± SD. P-values using student’s t-tests comparing Dox or Dox + MB versus control, *; *P* < 0.05 (No difference between control and MB only; Dox and Dox + MB among all parameters). ALP = Alkaline phosphatase, ALT = alanine aminotransferase, AST = aspartate aminotransferase, TBIL = Total Bilirubin, PHOS = phosphorus levels, CRE = Creatinine, BUN = blood urea nitrogen.

The unsatisfactory treatment outcome might be attributed to the low penetration and gradually dropping microbubble concentration within the UN-KC-6141 tumor (Fig. 1C,D). Additionally, the concentration of Dox in the mouse plasma was reported to decrease within 10 min after drug administration^34^. To improve the effect of sonoporation on drug penetration, an automatic pump for continuous infusion was used to maintain a constant influx of microbubbles into the tumor during the period of sonoporation, and the Dox was delivered simultaneously with microbubbles for sonoporation. A new protocol using the Power Doppler imaging mode was applied to this study (Fig. 2A,B) to explicitly target the tumor area for sonoporation and provide more pulses to increase the sonoporation effect^25^. The mice's survival result demonstrated that Power Doppler-based sonoporation significantly enhanced Dox efficacy by increasing the median surviving time from 27.0 to 33.5 days. The additional survival results of the mice from the treatment exhibited a threefold increase, from 3 days (Dox only) to 9.5 days (Dox + MB), compared to the control group with a survival time of 24.0 days. The 30-day survival rate increased from 30% in the Dox-only group to 50% in the Dox + MB group, with one mouse surviving for over six months (Fig. 2C). The ultrasound image showed that Dox + MB therapy could further inhibit tumor progression area than Dox monotherapy, resulting in 27% and 26% tumor area reduction on days 16 and 21, respectively (Fig. 2D,E). On the other hand, sonoporation only (MB only) didn’t affect tumor progression, and there was no significant difference in the mice survival compared to the control group (Fig. 2C,D,E). After treatment, the body weight of mice in both the Dox and Dox + MB groups experienced a slight 5% decrease, followed by a rapid recovery within three days. In contrast, the body weight of mice in the control and MB-only groups remained stable (Fig. 3A). To confirm if there were acute adverse reactions mediated by Dox or MB treatment, blood from tumor-bearing mice was collected and examined for biochemical values. The results revealed that only the Dox-related acute adverse reactions, such as liver inflammation (indicated by a significant increase in Alkaline Phosphatase (ALP) levels compared to the control), were observed in the Dox and Dox + MB groups. Additionally, a slight increase in phosphorus levels (PHOS) was noted after doxorubicin treatment, as compared to the control and MB-only group, though this difference was insignificant (Fig. 3B). There were no notable distinctions between the Dox and Dox + MB groups across all parameters. These data suggested that the Power Doppler-based NH002 sonoporation with Chemotherapy (PDNSC) was safe under this protocol and could significantly prolong the mice's survival by enhancing the efficacy of chemotherapy in pancreatic tumors.

### PDNSC altered the PDAC tumor microenvironment in favor of the drug effect

**Figure 4:**
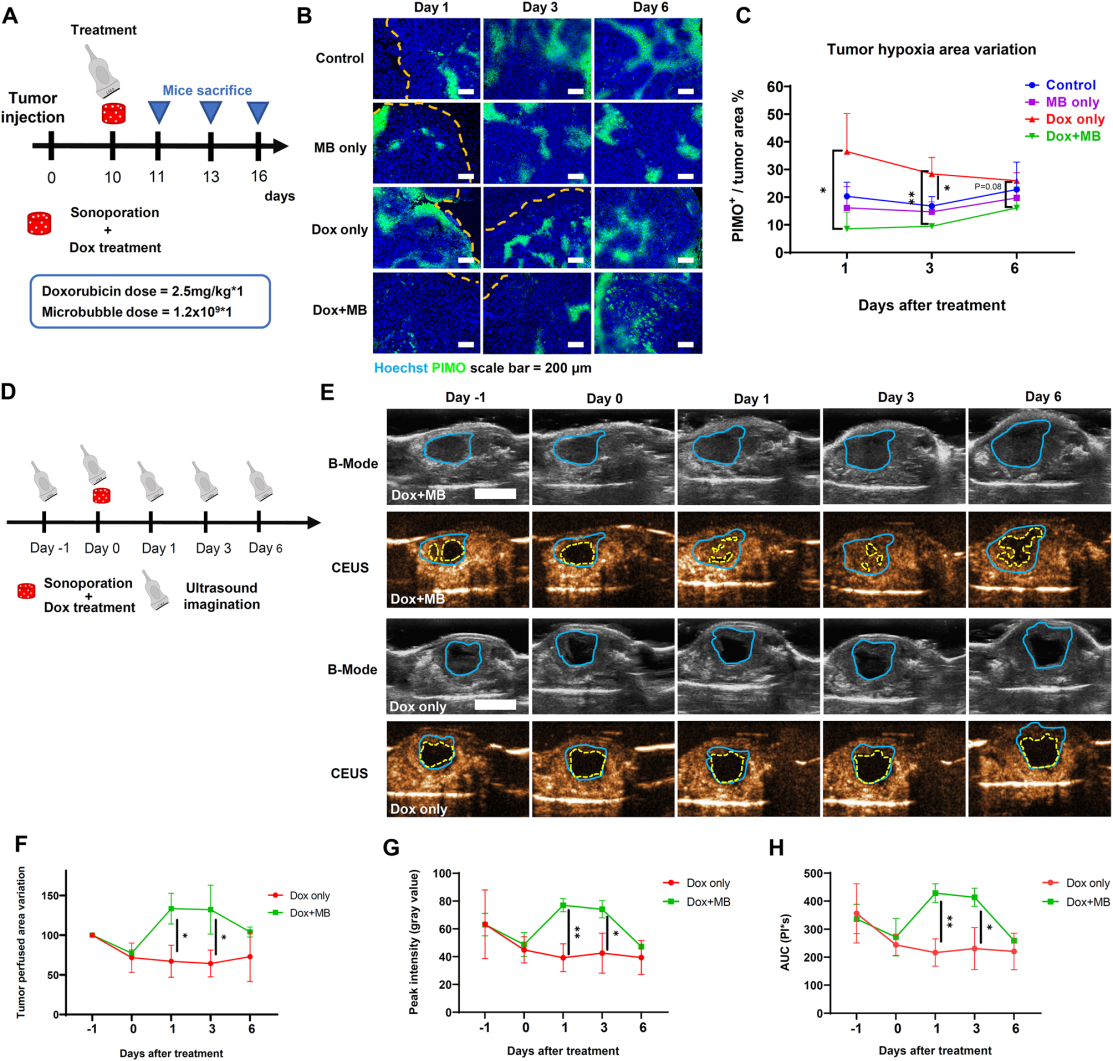
The change of UN-KC-6141 tumor microenvironment following Power Doppler-based sonoporation with NH002 and doxorubicin. (**A**) Experimental scheme. Ten days after tumor inoculation, mice were separated into four groups: Control, MB only, Dox only, and Dox + MB. Mice were sacrificed 1, 3, and 6 days after the treatment. Pimonidazole (160 mg/kg) was IP administered 1 h before sacrifice. (**B**) Tumor hypoxia regions were indicated with Pimonidazole staining (green), and nucleus cells were labeled with Hochest33342^+^ (blue). The yellow dashed line indicated the tumor border. Scale bar = 200 μm. (**C**) Quantification of hypoxia (Pimonidazole^+^) region (fraction of tumor area coverage). N ≧ 3 for each time point and group. (**D**) Perfusion examination experimental scheme. Perfusion test with TBS-002 microbubble was conducted − 1, 0, 1, 3, and 6 days after the treatment. Xenograft tumor-bearing mice were randomly separated into two groups when the tumor reached 50 mm^2^ under the ultrasound examination. The Dox only group received dox only, and Dox + MB group received dox plus Power Doppler-based sonoporation. (**E**) Representative images of the perfused tumor area. The tumor area (blue circle) was distinguished under the B-mode examination. Non-perfused area (yellow circle) was discerned under contrast mode as the dark area. Scale bar = 1 cm. (**F**) Statistics of tumor perfused area variation compared to the day before the treatment. (**G**) Quantitative of peak intensity variation in the tumor (blue circle). (**H**) Quantitative area under curve (AUC) variation in the tumor (blue circle). A two-tailed unpaired t-test was used to compare every two groups. *: *P* < 0.05, **: *P* < 0.01. N > 3 in each group.

According to the monitoring of tumor size, a significant reduction in tumor size was found at day 16 (the second treatment of Dox and sonoporation) and persisted until day 21 (Fig. 2D), suggesting a postponed effect than the direct enhancement of drug uptake reported in other sonoporation studies^21,35–37^. Therefore, it was hypothesized that the outcome might result from the altered TME after the sonoporation treatment. To examine whether our preclinical sonoporation setting triggered any changes in the TME, tumor tissues were collected on day 1, day 3, and day 6 after the first treatment and examined by immunofluorescence staining (Fig. 4A). Hypoxia in PDAC is an intrinsic factor causing resistance to chemotherapy^9^. We have previously reported that the UN-KC-6141 had low MVD and a high percentage of hypoxia^5^. The hypoxia status after treatments was further examined by staining the hypoxia probe, PIMO (Fig. 4B). The results showed that the Dox treatment significantly increased in the hypoxic area on day 1 and day 3 post-treatment compared to other groups. However, combination therapy led to significant decreases in hypoxia on day 1 (8.5% versus 36.5%) and day 3 (9.5% versus 28.4%) compared to Dox treatment. A slight decrease in the hypoxic region was also found on day 6 (16.1% versus 25.9%). However, it did not reach a statistical difference, indicating that the effect of combined treatment was sustained for at least 3 days and gradually attenuated 6 days after the first treatment (Fig. 4B,C). There was no statistical difference in the tumor hypoxic area between the control and the sonoporation-only groups in the observation window.

To further explore the effect of the PDNSC, CEUS was employed to continuously monitor variations in tumor perfusion over a week (Fig. 4D). Here, the xenograft model was used to achieve a better tumor delineation for precise quantification. The results indicated a significant increase in the variation of tumor perfusion area in the combination treatment group on day 1 (133% versus 67%) and day 3 (132% versus 64%) compared to the group that received Dox only (Fig. 4E and F). Both the peak intensity and the area under the curve (AUC) in the tumor were significantly higher on day 1 (approximately 1.9-fold higher) and day 3 (approximately 1.7-fold higher) in the combined treatment group than in the sole chemotherapy group (Fig. 4G and H). The increased tumor perfusion declined from day 3 to day 6, reaching similar levels of tumor perfusion, peak intensity, and AUC between the two groups (Fig. 4E–H). These data suggested that sonoporation with Dox significantly relieved the tumor's hypoxic status and increased tumor perfusion.

Gemcitabine has been the standard frontline drug for PDAC patients since 1997^6,38^. To assess the compatibility of our sonoporation setting with gemcitabine, we conducted an in vivo study (Fig. S3A) examining the combined effect of gemcitabine and sonoporation. Results showed that the combination treatment prolonged the survival of mice compared to those treated with gemcitabine alone (39.0 days versus 33.0 days, Fig. S3B). Ultrasound images revealed additional tumor growth inhibition in the combined treatment group on day 16 (33% tumor reduction) and day 21 (27% tumor reduction), compared to the gemcitabine-only group (Fig. S3C–D). Moreover, analysis of tumor hypoxia after treatment (Fig. S3E–G) demonstrated that combined sonoporation with gemcitabine significantly reduced the hypoxic area compared to gemcitabine alone (7.6% versus 33.4%). These findings indicate that PDNSC could combine therapeutic drugs, like Dox or gemcitabine, to modify the TME and enhance chemotherapeutic efficacy against pancreatic tumors.

### PDNSC strengthened the effect of αPDL1 immunotherapy

**Figure 5:**
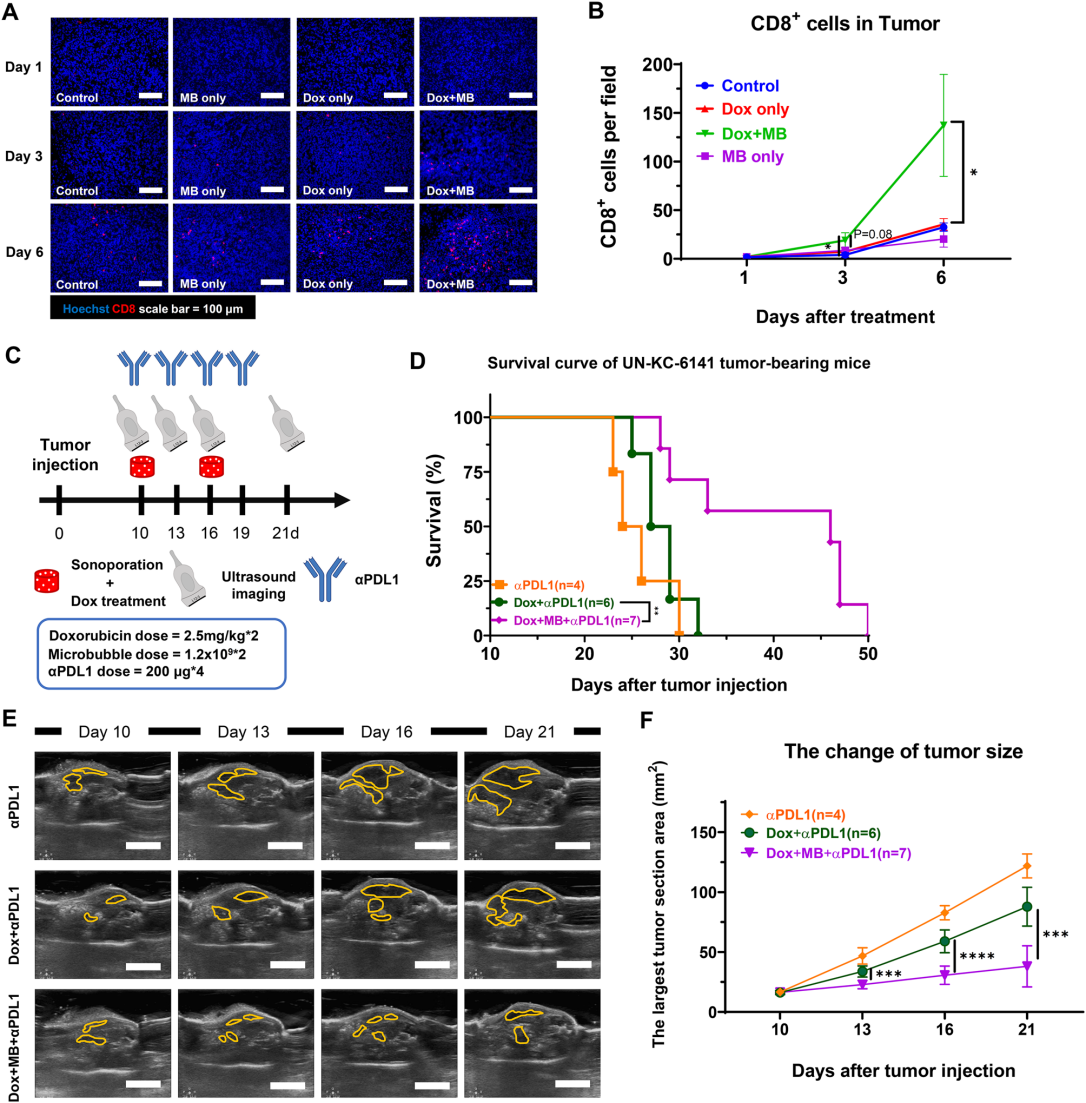
PDNSC strengthens the αPDL1 treatment and slows down UN-KC-6141 tumor progression. (**A**) Representative images of cytotoxicity T cells in the tumor area. Tumors were collected as depicted in Fig. 4A. Cytotoxicity T cells were indicated with CD8 staining (red). (**B**) Quantification of CD8^+^ T cells in the tumor region (per field). Scale bar = 100 μm. N ≧ 3 for each time point and group. (**C**) The schedule of the treatment protocol. The chemotherapy protocol was the same as the previous setting. The αPDL1 (8 mg/kg) was given on day10, 13, 16, and 21. (**D**) Kaplan–Meier survival curve of PDAC, αPDL1 group received αPDL1 only (n = 4), Dox + αPDL1 group received doxorubicin plus αPDL1 (n = 6). The Dox + MB + αPDL1 group received doxorubicin plus Power Doppler-based sonoporation and αPDL1 (n = 7). (**E**) Representative figures of ultrasound images (Yellow circle indicated tumor region) at different time points. (**F**) The largest tumor section area of orthotopic UN-KC-6141 tumor-bearing mice examined at 10, 13, 16, and 21 post-tumor implantation. Scale bar = 1 cm. A two-tailed unpaired t-test was used to compare the tumor size at each time point between the Dox + αPDL1 and Dox + MB + αPDL1 groups. *: *P* < 0.05, **: *P* < 0.01, ***: *p* < 0.001, ****: *p* < 0.0001. N > 3 in each group.

T cells act as a crucial modulator in the host immune system against tumors, extending the tumor eradication of chemotherapy. However, the T-cell infiltrating was strongly hindered by the harsh TME of PDAC^39^. To further test the effect of PDNSC on T-cell infiltration into tumors, the T cells within TME were identified using CD8 and CD4 markers on day 1, day 3, and day 6 following the initial treatment. The results demonstrated that both cytotoxic T cells (CD8^+^) and helper T cells (CD4^+^) were rarely found in control, Dox-only, and MB-only groups (Fig. 5A, S4A). Combined treatment resulted in a mild increase of CD8^+^ T cell infiltration on day 3 compared to control (*P* = 0.03), Dox- (*P* = 0.08), and MB-only groups (*P* = 0.09), but a profound expansion on day 6 with a 3.9-fold increase compared to any other groups (Fig. 5B). Infiltration of CD4^+^ T cells on day 6 with a 2.5-fold increase in the combined treatment group was also observed (Fig. S4B). The above results demonstrated that the PDNSC changed the TME of PDAC, decreasing hypoxia, enhancing tumor perfusion, and increasing T-cell infiltrates.

ICB therapy has emerged as a pivotal and promising treatment for cancer. However, it has not yet been proven effective for PDAC patients due to the harsh TME^40^. To further assess the potential for enhancing the efficacy of ICB therapy on PDACs through PDNSC, we administered the anti-PDL1 (αPDL1) antibody to tumor-bearing mice because our previous study had reported the high-level PDL1 expression on the UN-KC-6141 tumors^5^. Mice were administrated with αPDL1 on days 10, 13, 16, and 19, while the Dox and sonoporation treatment followed the previous schedule (Fig. 5C). The results revealed that triple combination treatment (Dox + MB + αPDL1) significantly prolonged the median survival of mice (46 days) compared to those of Dox + αPDL1 treatment (28 days) and αPDL1 only (25 days) (Fig. 5D). The triple combination therapy also significantly suppressed tumor growth compared to Dox + αPDL1 or αPDL1 alone (Fig. 5E and F). These data suggest that PDNSC has the potential to enhance the effectiveness of αPDL1 treatment and strengthen the combination of chemo-immunotherapy against pancreatic tumors.

## Discussion

The challenging prognosis of pancreatic ductal adenocarcinoma (PDAC) necessitates innovative theragnostic interventions to improve early detection and treatment efficacy. This study established a feasible method to detect the small lesions of pancreatic tumors and applied the Power Doppler-based sonoporation to enhance the chemotherapy and ICB therapy in an orthotopic murine UN-KC-6141 PDAC model. UN-KC-6141 tumor, like human PDAC, is a hypovascular tumor and does not well response to ICB therapy. Our results demonstrate that NH002 microbubble-based CEUS could effectively distinguish murine orthotopic pancreatic tumors (< 5 mm) from normal tissues. Moreover, this study illustrates the power of PDNSC to enhance chemotherapy efficacy and improve the effectiveness of ICB therapy. These results proved the potential of using a non-invasive NH002 microbubble for theragnostic value against the PDAC.

Ultrasound settings are crucial for successful sonoporation^41,42^. This study utilized the diagnostic ultrasound system with a broadband linear array transducer for all experiments, making it easily translatable to clinical practice. Initially, we employed B-mode-based sonoporation, referencing a clinical study^31^. However, it yielded minimal benefits for mice survival and tumor inhibition (Fig. S2C–G). Subsequently, the microbubble administration method was altered from four bolus injections to continuous infusion, ensuring microbubbles were perfused into the tumor's vessel network and generated on-site cavitation^43^. With sustained microbubble delivery, reperfusion of microbubbles to the tumor region could be achieved. Considering the different biological decay of Dox between humans and mice^34,44^, we co-administered Dox with NH002 microbubbles (Fig. 2). According to a previous study comparing different imaging modes, the Power Doppler mode could provide 15 more pulses in one transmission than the traditional B-mode^25^, thus increasing the cavitation doses in a regular diagnostic ultrasound system. Another advantage of the Power Doppler is the ROI selection. A doctor or sonographer can specifically target the tumor area for sonoporation without affecting nearby organs. Our study demonstrates that the above adjustments are critical to a successful treatment.

The MI value is another crucial factor in sonoporation. MI values below 0.2 result in linear microbubble oscillation, while values between 0.2 and 0.5 lead to nonlinear oscillation. Microbubble will expand and burst when MI exceeds 0.6^45^. According to the labeling of the marketed ultrasound contrast agents, such as Definity (Lantheus Medical Imaging, MA), in the absence of sufficient safety data, it is generally believed that microbubbles should not be used at mechanical indexes higher than 0.8^33^. Therefore, ensuring microbubbles generate significant oscillations and robust mechanical forces for observable therapeutic effects on surrounding cells or tissues while prioritizing safety is essential. In this study, we applied the intermediate mechanical index of 0.4 and observed no acute compliance issues in mice, as supported by the biochemistry test results (Fig. 3A,B). In summary, our study has demonstrated that the PDNSC is safe and can be performed to improve the efficacy of conventional chemotherapy using commercially available diagnostic ultrasound systems.

The highly proliferative tumor cells and inadequate blood supply lead to hypoxia within TME, thus hindering the delivery and therapeutic efficacy of chemical drugs^46^. Hypoxia in tumors becomes more severe after chemotherapy due to the toxicity of chemo-drugs, such as gemcitabine and doxorubicin, on endothelial cells, further compromising blood flow and reducing oxygen delivery^47,48^. Moreover, a study demonstrated that the TME becomes fibrotic after chemotherapy, potentially affecting oxygen diffusion and increasing the status of tumor hypoxia^49^. In the present study, UN-KC-6141 tumors showed an increasing trend of the hypoxic region as the tumor grew, with a further elevation of the hypoxic proportion in the tumors receiving Dox treatment (Fig. 4B,C). Tumor perfusion was also observed to decrease after Dox treatment. However, combining PDNSC with Dox significantly resulted in a nearly two-fold perfusion enhancement, reversing the upraised hypoxia area caused by doxorubicin treatment. Interestingly, the well-perfused status was sustained by combined therapy for at least six days after treatment and subsided after that. These data suggested that PDNSC could provide an optimal therapeutic window for combining with other adjuvant therapies, such as radiation therapy or ICB therapy, to enhance the efficiency of anti-tumor response.

ICB therapy has emerged as a viable and promising treatment for cancer by enhancing the function of T lymphocytes with monoclonal antibodies targeting the programmed cell death protein-1(PD-1), cytotoxic T lymphocyte-associated protein-4 (CTLA-4), and programmed death-ligand 1(PD-L1), various types of cancer patients benefited^40,50^. However, targeted T-cell therapy has been unsuccessful in PDAC patients due to the harsh TME of PDAC, preventing T cells from locating proximal enough to the cancer cells^51^. Remodeling the harsh TME has been studied to improve ICB therapy. Our previous study demonstrated that ablative radiation therapy increased perfusion and enhanced the efficiency of ICB therapy in the PDAC tumor model^5^. Similarly, a study utilized high-intensity focused ultrasound (HIFU) to treat neuroblastoma, which increased intratumoral NK cells, CD4, and CD8 T cells, overcoming therapeutic resistance to achieve long-term survival^52^. Additionally, Ningshan et al. employed ultrasound microbubble cavitation to enhance the perfusion of MC38 colon cancer, thus improving the effectiveness of αPDL1 treatment and highlighting the significance of modifying TME as an adjuvant strategy to improve current existing therapy^53^. The present study demonstrated that PDNSC modulated the TME of PDAC to lesser hypoxia, better perfusion, and more T-cell infiltration. These alterations in TME consequently increase the efficacy of αPDL1 therapy. This study proved the feasibility of enhanced ICB treatment by PDNSC and provided a novel strategy for clinics to treat PDAC tumors.

Various cocktail chemo-drugs with or without adjuvant therapy for PDAC patients have been intensely tested and studied^54,55^. This study proved a specific sonoporation setting using PDNSC that can reduce tumor hypoxia and enhance the efficacy of doxorubicin and gemcitabine for pancreatic tumors. However, to consider the application of this treatment strategy in clinical practice, other promising newly released drug interventions for PDAC patients, such as gemcitabine plus nab-paclitaxel and FOLLFIRINOX (5-FU, irinotecan, folinic, oxaliplatin), should be assessed in the future. The present study administered two doses of drugs to tumor-bearing mice, and the sonoporation time for the NH002 microbubble was set to 4 min for each sonoporation treatment in the murine PDAC model. The dosages and sonoporation protocol should also be optimized to adapt to current clinical practice, such as longer sonoporation time or shorter chemotherapy intervals. In this study, the administration of doxorubicin or gemcitabine combined with sonoporation significantly reduced hypoxia, whereas sonoporation alone did not exhibit such a pronounced effect. These findings suggest that the two chemo-drugs might synergize with Power Doppler-based NH002 sonoporation to increase tumor perfusion and reduce the tumor hypoxic region. Hence, we recommend delivering the tested chemo-drugs simultaneously with sonoporation to achieve the best outcome. However, whether the synergistic effect of sonoporation applies to other chemical drugs should be further examined.

Finally, several uncertainties warrant further investigation to facilitate the translation of our findings into clinical practice. Firstly, while our study utilized an orthotopic PDAC model, enhancing human relevance necessitates exploration using human pancreatic cancer cell lines or patient-derived models. Secondly, our study employed uniform doses of microbubbles and drugs across all in vivo experiments; thus, future investigations should include dose-ranging studies to assess efficacy and potential toxicities. Thirdly, while we evaluated an MI value of 0.4 for microbubble sonoporation, exploration of other ultrasound parameters for comparable or enhanced benefits is warranted. Further investigations into the optimal ultrasound settings are crucial for maximizing therapeutic outcomes.

## Conclusion

This study demonstrated that NH002 microbubble-based CEUS via diagnosed ultrasound equipment can detect early-stage PDAC tumors and enhance the therapeutic effect of chemotherapy in a murine orthotopic PDAC model. The PDNSC setting reduced the hypoxia status, elevated the perfusion of tumors, and increased T-cell infiltration. In addition, combined PDNSC with ICB therapy retarded tumor growth effectively. Overall, this novel modality could improve the treatment outcomes for PDAC patients and encourage future efficacy and safety evaluations in clinical trials.

### Supplementary Information

Supplementary Information 1.

Supplementary Video 1.

Supplementary Figures.

## Data Availability

The datasets generated and analyzed during the current study are available from the corresponding author upon reasonable request.
