# Supplementary material for: Power-Doppler-based NH002 microbubble sonoporation with chemotherapy relieves hypoxia and enhances the efficacy of chemotherapy and immunotherapy for pancreatic tumors
Source: Sci Rep. 2024 Jun 3;14:8532. doi: 10.1038/s41598-024-54432-y (PMC11148017; doi:10.1038/s41598-024-54432-y)
Supplement: Supplementary file 1 — Supplementary Information 1. [file 41598_2024_54432_MOESM1_ESM.docx]

Supplement video 1: The time-lapse video of tissue perfusion after microbubble injection corresponds to Figure1B-D.
