# Supplementary material for: Power-Doppler-based NH002 microbubble sonoporation with chemotherapy relieves hypoxia and enhances the efficacy of chemotherapy and immunotherapy for pancreatic tumors
Source: Sci Rep. 2024 Jun 3;14:8532. doi: 10.1038/s41598-024-54432-y (PMC11148017; doi:10.1038/s41598-024-54432-y)
Supplement: Supplementary file 3 — Supplementary Figures. [file 41598_2024_54432_MOESM3_ESM.pdf]

Power-Doppler-based NH002 microbubble Sonoporation with Chemotherapy Relieves Hypoxia and Enhances the efficacy of Chemotherapy and Immunotherapy for Pancreatic Tumors

Sheng-Yan Wu[1], Chung-Hsin Wang[2], Shih-Tsung Kang[2], Ching-Fang Yu[3,4], Fang-Hsin Chen[5,6] and Chi-Shiun Chiang[1,5,6]

Supplement figure 1

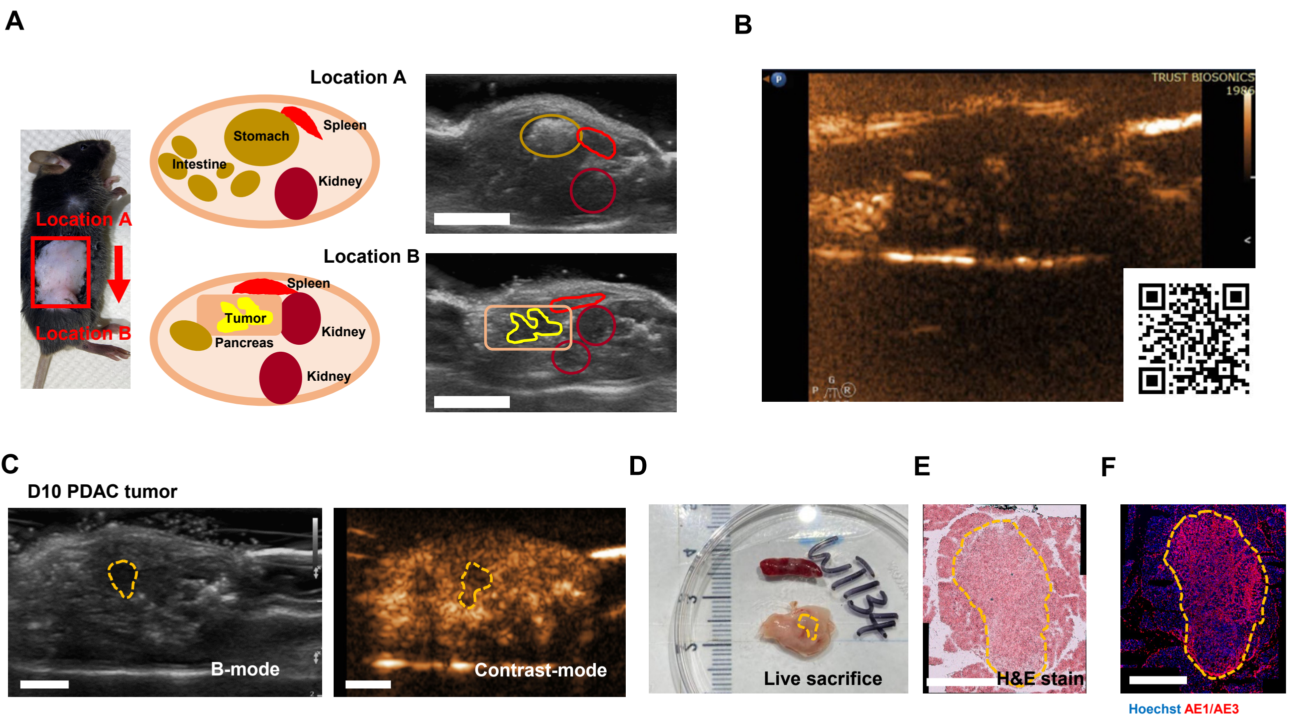

Supplement Figure 1. Characterization of small murine UN-KC-6141 PDAC tumor. (A) The ultrasound imaging was performed from locations A to B. The scheme was shown as relative organ position changing when performing an ultrasound examination. Brown circle (Stomach), red irregular circle (Spleen), dark red circle (Kidney), skin circle (Pancreas), and yellow irregular circle (Tumor). (B) The time-lapse video of tissue perfusion after microbubble injection corresponds to Figure 1B-D. Video link: https://youtu.be/ItcObjoPcIA. (C) The ultrasound image of day10 tumor-bearing mice, B-mode image (left) and CEUS image (right), yellow circle = tumor. Scale bar = 0.5 cm. (D) Tissue image after mice were euthanized (Yellow circle = tumor). (E) H & E staining of pancreas tissue with tumor (blue circle). (F) Immunofluorescence staining of AE1/AE3 (red) on tumor tissue. Scale bar = 0.1 cm

Supplement figure 2

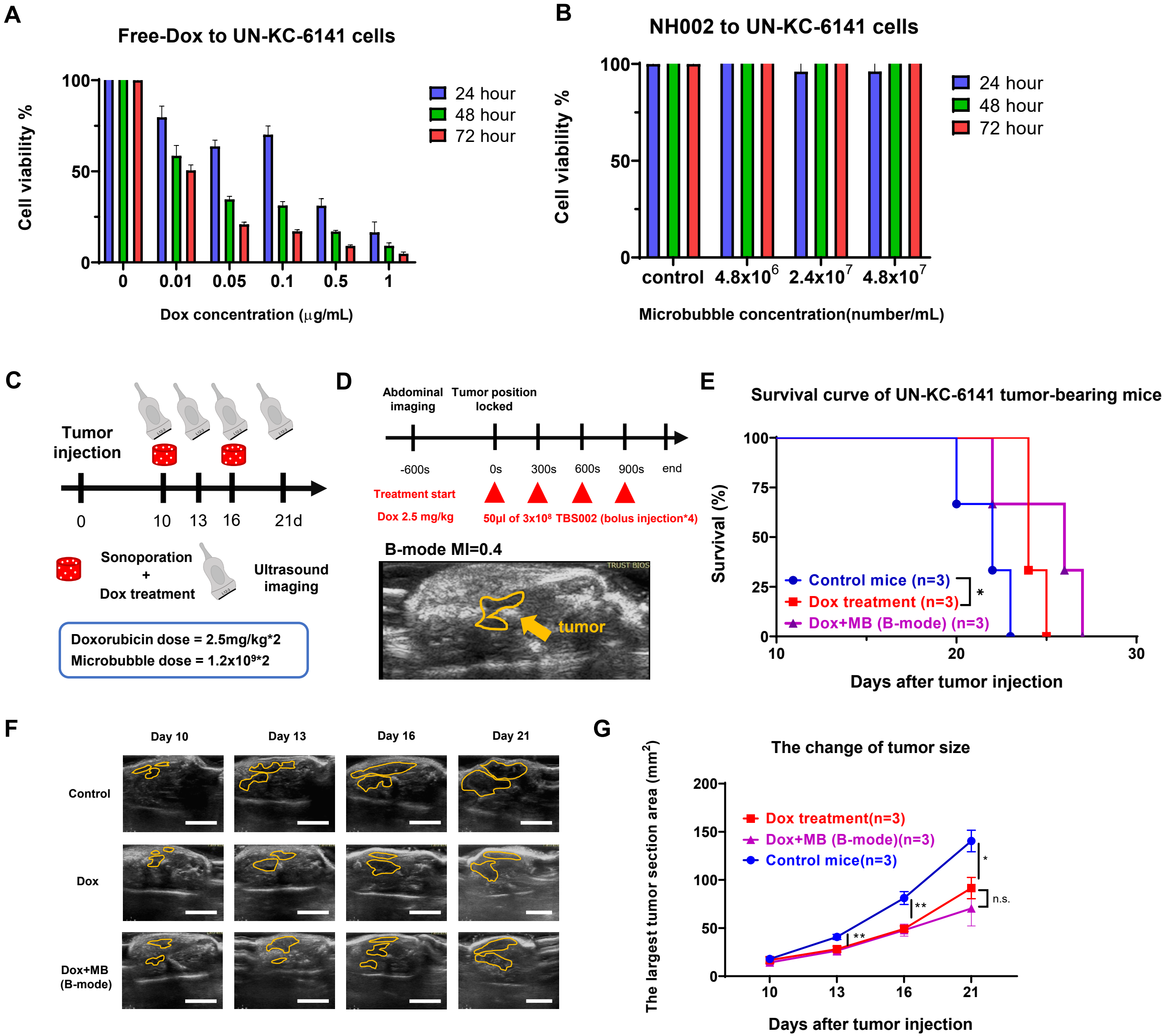

Supplement Figure 2. Effect of doxorubicin treatment and B-mode sonoporation on UN-KC-6141 tumor-bearing mice. (A, B) Cytotoxicity test of doxorubicin and NH002 microbubble on UN-KC-6141 cells. Different concentration of dox or bubble was given to cells for 24, 48, and 72 hours and tested by the microplate reader. (C) The scheme of a treatment protocol. Ten days after implantation of UN-KC-6141 cells. Mice were randomly grouped, and the treatment mice were bolus injected two times (at a 6-day interval) with 2.5 mg/kg doxorubicin. Ultrasound imagination of tumor size was examined at 10, 13, 16, and 21 days post-implantation. Sonoporation was performed at the same time as Dox administration. (D) B-mode sonoporation protocol. Dox was given 10 minutes before sonoporation. 50 µl of 3 x 10^8 microbubbles were given to mice every 5 minutes four times, and the total sonoporation time was 20 minutes. (E) Kaplan-Meier survival curve of UN-KC-6141 tumor-bearing mice, Control mice without any treatments (n=3), Dox only group receive doxorubicin only (n=3), and Dox+MB group receive doxorubicin plus B-mode-based sonoporation (n=3). (F) Representative figures of ultrasound images (Yellow circle indicated tumor area) at different time points. (G) Quantification of the largest tumor section area of orthotopic UN-KC-6141 tumor-bearing mice. Scale bar = 1 cm. A two-tailed unpaired t-test was used to compare the tumor size between the control and dox groups at each time point. *: $P < 0.05$, **: $P < 0.01$.

Supplement figure 3

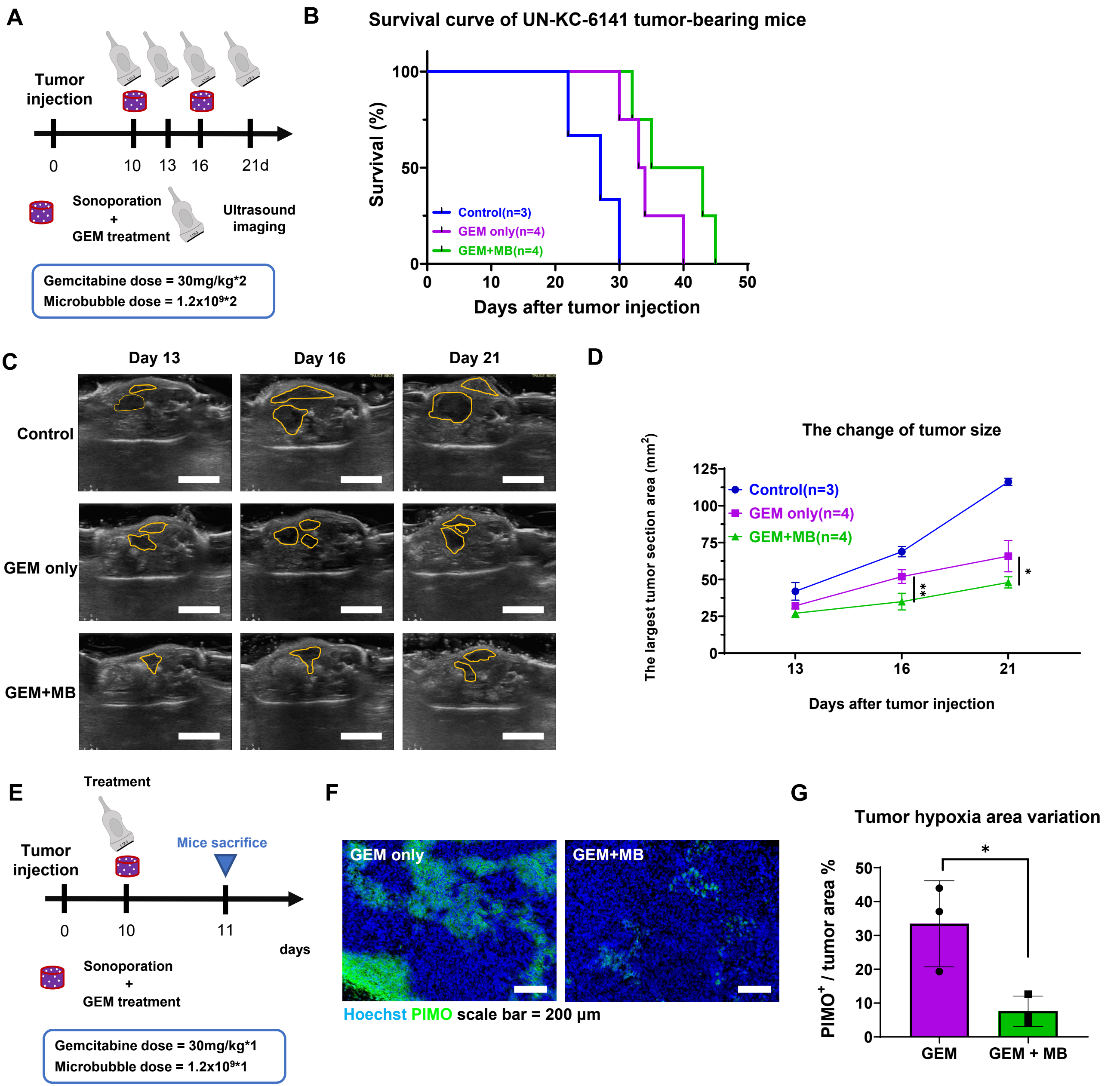

Supplement Figure 3. Combined gemcitabine with Power Doppler-based sonoporation slows down UN-KC-6141 tumor progression. (A) The schedule of the treatment protocol. (B) Kaplan-Meier survival curve of PDAC, Control mice without any treatments (n=3), GEM-only group received gemcitabine only (n=4), GEM+MB group received gemcitabine plus Power Doppler-based sonoporation (n=4). (C) Representative figures of ultrasound images (Yellow circle indicated tumor region) at different time points. (D) The largest tumor section area of orthotopic UN-KC-6141 tumor-bearing mice examined at 10, 13, 16, and 21 post-tumor implantation. Scale bar = 1 cm. (E) The experimental scheme. Ten days after tumor inoculation, mice were separated into two groups, GEM only (n=3), and GEM+MB (n=3). Mice were sacrificed 1 day after the treatment. Pimonidazole (160 mg/kg) was i.p. administered 1 hour before sacrifice. (F) Representative images of hypoxia (Pimonidazole^{+}, shown in green) area in tumor. Scale bar = 200 μm. (G) Quantitative data of hypoxia area in the tumor. A two-tailed unpaired t-test was used to compare the tumor size at each time point between the GEM-only and GEM+MB groups. *: $p<0.05$, **: $p<0.01$.

Supplement figure 4

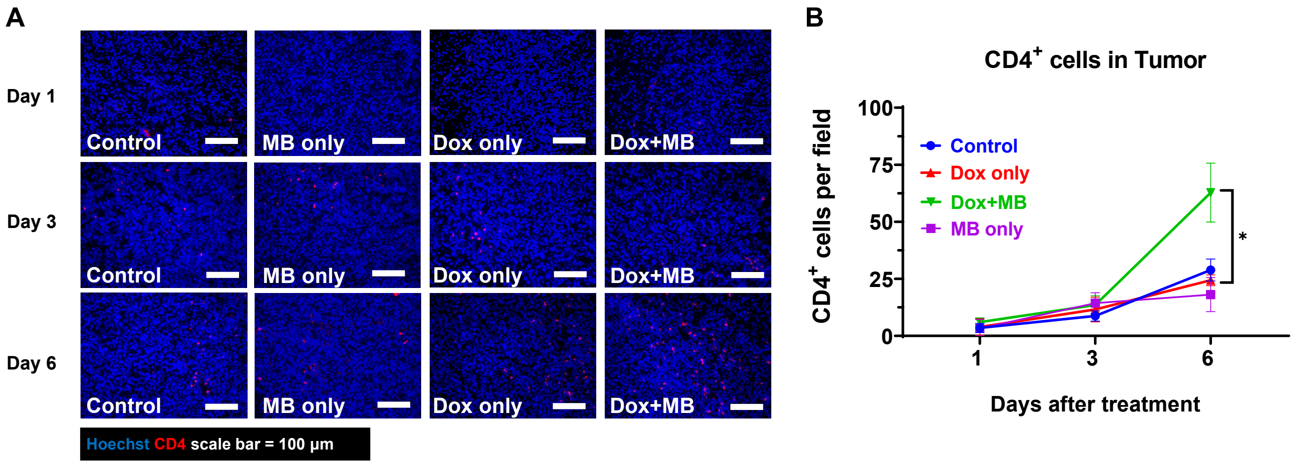

Supplement Figure 4. The change of CD4 T cells in tumors following PDNSC. (A) Representative images of CD4 T cells in the tumor area. Tumors were collected as depicted in Figure 3A. CD4 T cells were indicated with CD4 staining (red). Scale bar = 100 µm. (B) Quantification of $CD4^+$ T cells in the tumor region (per field). A two-tailed unpaired t-test was used to compare every two groups. *: P <0.05, **: P <0.01. N>3 in each group.
